# Multidrug Resistance Gram-negative Bacteria in Intensive Care Unit of Tertiary Care Hospital: A Descriptive Cross-sectional Study

**DOI:** 10.31729/jnma.8747

**Published:** 2024-09-30

**Authors:** Raina Chaudhary, Sabita Bhatta, Chiranjibi Pant, Naveen Phuyal, Lochana Shrestha

**Affiliations:** 1 Department of Microbiology, Nepalese Army Institute of Health Sciences, Sanobharyang, Kathmandu, Nepal; 2 Department of Medicine, Nepalese Army Institute of Health Sciences, Sanobharyang, Kathmandu, Nepal; 3 Department of Community Medicine, Nepalese Army Institute of Health Sciences, Sanobharyang, Kathmandu, Nepal

**Keywords:** *acinetobacter*, *gram negative bacteria*, *intensive care unit*, *multi drug resistance*

## Abstract

**Introduction:**

Antimicrobial resistance is global health problem, amongst major causes for mortality. It is one of the hinderance for achievement of Sustainable Goal 3 (Good health and well-being) of World Health Organization. Multidrug resistant gram negative bacteria are major threat to humanity especially patients admitted in intensive care unit. This is associated with to treatment failure and mortality of the patients in intensive care unit. Therefore, this study was conducted to find out the prevalence of multidrug resistant gram negative bacteria in intensive care unit of tertiary care center.

**Methods:**

A descriptive cross-sectional study was conducted in the Department of Microbiology of a tertiary care hospital from February, 2020 to August, 2021 for 18 months after obtaining ethical approval from the Institutional Review Committee (Reference Number: 246). All the samples from Intensive Care Unit were processed following standard methodology. Only gram-negative bacteria isolated from samples were included in the study. Convenience sampling method was used. The point estimate was calculated at 95% Confidence Interval. Data was entered in Microsoft Excel 2016 and analysis was done using IBM SPSS Statistics version 16.0.

**Results:**

Out of 500 samples, 380 (76%) showed growth of gram negative bacteria. The prevalence multidrug resistance was 316 (83.15%). The multidrug resistant bacteria isolates were *Acinetobacter* species 43 (100%), non fermenter 57 (95%) and *Escherichia coli* 129 (87.16%). Multidrug resistant isolates were least resistant towards carbapenem group of antibiotics.

**Conclusions:**

The prevalence of multidrug resistant gram negative bacteria was found to be similar to other studies. Susceptibility towards commonly used both oral and intravenous antibiotics was low.

## INTRODUCTION

Antimicrobial resistance (AMR) is top ten global health problem and threats for humanity. According to World Health Organization (WHO), AMR was responsible for 1.27 million global deaths in 2019. A multisectoral approach is required to combat AMR for the achievement of the Sustainable Development Goals-3.^1^ Multi drug resistance (MDR) is defined as acquired non-susceptibility to at least one agent in three or more antimicrobial categories.^2^ MDR Gram negative bacteria (GNB) are usually resistance to broad spectrum antibiotics like third generation cephalosporin, fluroquinolone, aminoglycosides and carbapenem group.^3^

The MDR GNB are commonly responsible for infections in Intensive Care Unit (ICU) patients with invasive devices.^4^ It is recommended to continually refine standards on antimicrobial susceptibility.^3^ Therefore, this study was conducted to find out prevalence of MDR GNB from ICU samples and to establish guidance for empirical antibiotic therapy of critical patients.

## METHODS

A descriptive cross-sectional study was conducted in Department of Microbiology, after taking ethical clearance from Institutional Review Committee (IRC) (reference number 246). This study was conducted from February, 2020 till August, 2021 for 18 months. Total sampling method was used and 500 samples were included in study period.

All the samples including urine, pus & pus aspirate, lower respiratory tract samples; sputum, bronchoalveolar lavage, blood, sterile body fluids; cerebrospinal fluid, pleural fluid, peritoneal fluid, joint aspirate and urogenital tract swabs received in laboratory were processed following standard methodology. Urine samples were inoculated in Cysteine Lactose Electrolyte Deficient (CLED) media while other samples were inoculated in Blood Agar, MacConkey Agar and Chocolate Agar media. Gramnegative bacteria non repetitive samples were included whereas Gram positive bacteria were excluded in the study.

Sample showing pure growth of Gram-negative bacteria were processed for further identification. Various biochemical test like Catalase test, Oxidase test, Triple Sugar Iron (TSI) Agar test, Sulphur Indole Motility (SIM) test, Simmons Citrate Agar test, Christensen Urea Agar test, Methyl Red & Voges Proskauer test, Amino acid decarboxylase test, Oxidative-Fermentative (Hugh & Leifson's) test were performed. Antimicrobial Susceptibility Testing (AST) was carried out by Modified Kirby Bauer disk diffusion method. Various antibiotic like Ampicillin (10μg), Amoxicillin+clavulanate (20/10μg), Piperacillin (100μg), Pipperacillin+tazobactum (100/10μg), Cefixime (5μg), Cefotaxime (30μg), Cefipime (30μg), Ceftazidime (30μg), Amikacin (30μg), Gentamycin (10μg), Ciprofloxacin (5μg), Ofloxacin (5μg), Doxycycline (30μg), Trimethoprim/sulfamethoxazole (1.25/23.75μg), Imipenem (10μg) and Meropenem (10μg) were used. Interpretation of AST was done as per Clinical and Laboratory Standards Institute (CLSI) guideline 2020.^4^

Antibiotic discs from Hi Media Laboratories Pvt. Ltd., India were used. Antibiotic disc was checked for their lot number, manufacture, and expiry date with storage condition. For standardization of the Kirby-Bauer disc diffusion test, performance testing of antibiotics and Muller Hinton Agar (MHA) was done by using Escherichia coli ATCC 25922 and Pseudomonas aeruginosa ATCC 27853 as quality control strains. Quality of sensitivity tests was maintained by maintaining the thickness of MHA at 4mm and pH at 7.2-7.4. Quality control of Laboratory equipment, reagents, and media was carried out on a regular basis.

Data was entered in Microsoft Excel 2016 and analysis was done using IBM SPSS Statistics version 16.0. The point estimate was calculated at a 95% CI.

## RESULTS

A total of 500 different samples from ICU were included in the study among which 451 (90.2%) samples had shown growth of organism. Out of all growth, 380 (84.26%) were Gram-negative bacteria (Table 1).

**Table 1:** Distribution of Gram-negative bacteria in various samples (n = 380)

S. No.	Organisms	Pus & Pus Aspirate	Urine	Lower Respiratory Tract	Blood	Sterile Body Fluid	Urogenital tract
1.	*Escherichia coli*(n=148)	31 (20.94)	92 (62.16)	15 (10.13)	4 (2.70)	4 (2.70)	2 (1.35)
2.	Non fermenters (n=60)	31 (51.66)	7 (11.66)	10 (16.66)	10 (16.66)	1 (1.66)	1 (1.66)
3.	*Acinetobacter species* (n=43)	23 (53.48)	3 (6.97)	3 (6.97)	14 (32.55)	-	-
4.	*Klebsiella pneumoniae* (n=38)	15 (39.47)	3 (7.89)	14 (36.84)	5 (13.15)	-	1 (2.63)
5.	*Enterobacter species* (n=37)	18 (48.64)	3 (8.10)	15 (40.54)	-	-	1 (2.70)
6.	*Pseudomonas aeruginosa* (n=54)	13 (24.07)	11 (20.37)	21 (38.88)	8 (14.81)	-	1 (1.85)
	Total (n=380)	131 (34.47)	119 (31.31)	78 (20.52)	41 (10.78)	5 (1.31)	6 (1.57)

The prevalence of MDR was 316 (83.15%). Distribution of MDR gram negative bacteria were as follows: *Escherichia coii*, 129 (87.16%); Non fermenters, 57 (95%); *Acinetobacter species*, 43 (100%); *Klebsiella pneumoniae*, 32 (84.21%); *Enterobacter species*, 30 (81.08%), *Pseudomonas aeruginosa*, 25 (46.29%). Resistance pattern of *Escherichia coii* was 129 (100%) for Ampicillin, 129(100) for Cefixime and 129 (100%) for Ciprofloxacin. *Acinetobacter species* was 57 (100%) resistant to Ampicillin and other frequently used oral antibiotic (Table 2).

**Table 2:** Resistant pattern of MDR Gram negative bacteria (n= 316).

Antibiotics	*Escherichia coli* (n=129)	Non-fermenter (n=57)	*Acinetobacter* species (n=43)	*Klebsiella* species (n=32)	*Enterobacter* species (n=30)	*Pseudomonas aeruginosa* (n=25)
Ampicillin	129 (100)	57 (100)	NT	32 (100)	30 (100)	NT
Piperacillin	NT	NT	NT	NT	NT	25 (100)
Amoxicillin + Clavulanate	110 (85.27)	57 (100)	NT	32 (100)	30 (100)	NT
Pipperacillin + Tazobactum	81 (62.79)	48 (84.21)	43 (100)	13 (40.62)	19 (63.33)	12 (48)
Cefixime	129 (100)	57 (100)	NT	32 (100)	30 (100)	NT
Cefotaxime	76 (58.91)	57 (100)	43 (100)	32 (100)	30 (100)	NT
Cefipime	NT	NT	43 (100)	NT	NT	11 (44)
Ceftazidime	NT	NT	43 (100)	NT	NT	25 (100)
Amikacin	52 (40.31)	33 (57.89)	38 (88.37)	12 (37.5)	16 (53.33)	12 (48)
Gentamicin	48 (37.20)	32 (56.14)	40 (93)	16 (50)	18 (60)	10 (40)
Ciprofloxacin	129 (100)	57 (100)	43 (100)	32 (100)	30 (100)	25 (100)
Ofloxacin	101(78.29)	57 (100)	43 (100)	28 (87.50)	25 (83.33)	18 (72)
Doxycycline	59 (45.73)	57 (100)	-	24 (75)	21 (70)	NT
Trimethoprim/sulfamethoxazole	84 (65.11)	43 (75.43)	36 (83.72)	24 (75)	30 (100)	NT
Imipenem	24 (18.60)	37 (64.91)	35 (81.39)	11 (34.37)	15 (50)	10 (40)
Meropenem	26 (20.15)	39 (68.42)	35 (81.39)	11 (34.37)	15 (50)	08 (32)

NT: Not Tested, MDR: Multidrug resistance

## DISCUSSION

Several studies conducted from the different institution from Nepal as well as different part of the world stated that prevalence of the MDR Gram negative bacteria is increasing over the time. The prevalence ranges from 54% to 95.8%.^4-7^ In our study we found prevalence of MDR GNB 83.15%. It was almost similar with study conducted by Parajuli et al. (95.8%), Silpakar et al .(91.3%) from Nepal.^6-7^

Prevalence of such bacteria is much higher in ICU setting due to the various reasons like selective pressure of broad-spectrum antibiotics; Aminoglycosides, Azithromycin, Carbapenems, third & fourth generation Cephalosporins, Doxycycline, Quinolones and Piperacillin/tazobactam, invasive devices; intravenous catheters, urinary catheters, ventilator co-morbid states; hypertension, diabetes, cardiac diseases, vascular diseases, pulmonary diseases, chronic kidney diseases, serum positive for HIV/Hepatitis B & C, Cancer, Behavioral disorders, leukemia and autoimmune disorders, prolong hospital stay and lack of proper infection prevention & control practices. At the same time, non-therapeutic use of antibiotics in human, animal and agriculture are major contributors for MDR.

In this study prevalence of MDR was notably higher among *Acinetobacter* spp (100%). Previously, the prevalence of *Acinetobacter* was reported as 67.83% from same institute.^8^ The reason behind higher prevalence of MDR in our study might be due to inclusion of critical units' sample only. This is indicating that critical patients are at risk of acquiring MDR GNB in ICU. Other institute from different part of the Nepal are also no more exception, they had reported prevalence of *Acinetobacter* as follows Rajbhandari et al. (84.6%), Khanal et al. (85.4%), Yadav et al. (91%), Parajuli et al . (94.2%), which all were significant.^4,6,9,10^

*Acinetobacter* species has ability to survive in the environment for a prolonged period, even in dry conditions on particles and dust. Various studies had proven that environmental contamination result into the rapid spread of MDR Acinetobacter species.^11^

In this study, *Acinetobacter* species had shown less resistance towards Imipenem (81.39%) which was similar with the finding of studies conducted from different part of the world; Soni et al. (79.3%), Yadav et al. (79.7%), Siwakoti et al. (81%) and Parajuli et al. (86.4%).^6,9,12,13^ These finding states that still there is a hope for treatment of MDR *Acinetobacter* spp with Imipenem. However, in some of the studies it had been stated drug of choice for MDR *Acinetobacter* as Colistin Sulphate.^6,12,14^

*Pseudomonas aeruginosa* is one of the most frequently isolated organisms from inpatient. It is often associated with higher mortality compared to other GNB. In the The Extended Prevalence of Infection in Intensive Care (EPIC II) study *Pseudomonas aeruginosa* was found to be 19.9% of infection in ICU.^15^ However, in our study *Pseudomonas aeruginosa* were least isolated organism (46.29%). All *Pseudomonas aeruginosa* had shown 100% resistant towards third generation antipseudomonal cephalosporin (Ceftazidime). Of all Imipenem (40%) and Meropenem (32%) resistant. Study conducted by Yadav SK et al. had shown resistance of Imipenem (57.8%) which was almost similar whereas Meropenem (61.5%) was contrary to our study.^9^

In this study *Escherichia coli* was most frequently isolated Gram-negative bacteria from urine sample. The MDR *Escherichia coli* was (87.16%) which was almost similar with the study conducted by Mania et al. (90%).^16^ In our study *Escherichia coli* had shown least resistant towards Imipenem (18.60%) & Meropenem (20.15%) followed by Gentamicin (37.20%). However, absolute resistant was observed among commonly used oral antibiotic like Ciprofloxacin. This might be due to easy access to pharmacy for self-medication practice.

In 2017, WHO developed the first Bacterial Priority Pathogen List (BPPL) which includes bacterial pathogen of public health importance to guide research, development and strategies to prevent and control antimicrobial resistance. According to this guideline, Antibiotic Resistance (ABR) bacterial pathogens that pose the highest threat to public health due to limited treatment options, high disease burden (mortality and morbidity) and increasing trends in ABR, with few or no promising candidates in the pipeline are considered as critical group of pathogens. Infections with such isolates may be uniquely difficult to prevent and are highly transmissible; the pathogens may have global mechanisms of resistance and/or MDR strains in certain populations like critically ill personnels. Carbapenem resistant Enterobacterales, third generation Cephalosporin resistant Enterobacterales and Carbapenem-resistant *Acinetobacter* species are included in critical group.^17^

In this study, Carbapenem resistant Enterobacterales were noticeable. *Enterobacter* species (50%) and *Klebsiella pneumoniae* (34.37%) had shown equal resistance towards both Imipenem & Meropenem. However, *Escherichia coli* had shown different resistance percentage Imipenem(18.60%) and Meropenem (20.15%) . Third generation Cephalosporin resistant Enterobacterales are also notably high. In this study, *Klebsiella pneumoniae* and *Enterobacter* species had shown 100% resistance towards both Cefixime & Cefotaxime whereas *Escherichia coli* were 100% resistant to Cefixime while 58.91% resistant to Cefotaxime.

Likewise, prevalence of Carbapenem-resistance in *Acinetobacter* species was 81.39% for both Imipenem & Meropenem. This finding was almost similar with a Nepalese study conducted by Yadav et al. Imipenem (81.9%) and Meropenem (79.7%).^13^

This was single centric study with small sample size. Only critically ill patients were included. Sensitivity of Colistin Sulphate could not be performed as it had to be performed by Minimum Inhibitory Concentration (MIC) method as mentioned in CLSI guideline. The effectiveness of antibiotics could not be monitored after dispatch of final report. Both phenotypic and genotypic mechanism of antibiotic resistance could not be studied.

## CONCLUSION

The prevalence of the MDR GNB was found to be similar with other studies. Susceptibility towards commonly used both oral and intravenous antibiotics was low. Nevertheless, MDR GNB had shown reassuring effectiveness towards carbapenem group of antibiotics.
